# Supplementary material for: Nanocarriers Containing Curcumin and Derivatives for Arthritis Treatment: Mapping the Evidence in a Scoping Review
Source: Pharmaceutics. 2025 Aug 6;17(8):1022. doi: 10.3390/pharmaceutics17081022 (PMC12388981; doi:10.3390/pharmaceutics17081022)
Supplement: Supplementary file 1 [file pharmaceutics-17-01022-s001.zip › Sato et al., 2025 - Supplementary Files.pdf]

Supplementary file

Nanocarriers Containing Curcumin and Derivatives for Arthritis Treatment: Mapping the Evidence in a Scoping Review

Beatriz Yurie Sugisawa Sato[1#], Susan Iida Chong[1#], Nathalia Marçallo Peixoto Souza[2], Raul Edison Luna Lazo[2], Roberto Pontarolo[2], Fabiane Gomes de Moraes Rego[2], Luana Mota Ferreira[2], Marcel Henrique Marcondes Sari[2*]

[1]Departamento de Análises Clínicas, Curso de Biomedicina, Universidade Federal do Paraná, Curitiba, 80210-170, Paraná, Brasil.

[2]Programa de Pós-Graduação em Ciências Farmacêuticas, Universidade Federal do Paraná, Curitiba, 80210-170, Paraná, Brasil.

[#]These authors are designated as co-first authors, reflecting their equivalent contributions in this study.

*Correspondence author

Marcel Henrique Marcondes Sari

E-mail: marcelsari@ufpr.br

Tables

Table S1. Search strategies for Pubmed, Scopus, and Web of Science articles.

Database	Queries
**Pubmed**	((((((((((((((((nanoparticle[MeSH Terms]) OR (nanocapsule[MeSH Terms])) OR (nanogel[MeSH Terms])) OR nanosphere[MeSH Terms])) OR ("nanoparticle drug delivery system"[MeSH Terms])) OR (drug carrier[MeSH Terms])) OR ("solid lipid nanoparticle*"[Title/Abstract])) OR ("nanostructured lipid carrier*"[Title/Abstract])) OR (nanoemulsion*[Title/Abstract])) OR (microemulsion*[Title/Abstract])) OR (nanocarrier*[Title/Abstract])) OR ("lipid nanoparticle*"[Title/Abstract])) OR ("nanostructured system*"[Title/Abstract])) OR (liposome*[Title/Abstract])) OR (ethosome*[Title/Abstract])) OR (niosome*[Title/Abstract])) OR (cyclodextrin*[Title/Abstract]) AND (((Arthritis[MeSH Terms])) OR ("Rheumatoid arthrit"[Title/Abstract])) OR (arthrit*,[Title/Abstract] AND rheumatoid[Title/Abstract])) OR (osteoarthrit*[Title/Abstract])) OR (arthrit*,[Title/Abstract] AND degenerative[Title/Abstract])) OR ("Degenerative Arthrit*"[Title/Abstract])) OR ("Osteoarthros* Deformans"[Title/Abstract])) OR ("Spondyloarthrit* Ankylopoietica"[Title/Abstract])) OR ("Ankylosing Spondylarthrit*"[Title/Abstract])) OR (spondylarthrit*,[Title/Abstract] AND ankylosing[Title/Abstract])) OR ("Ankylosing Spondylitis"[Title/Abstract])) OR ("Spondylarthrit* Ankylopoietica"[Title/Abstract])) OR ("Bechterew Disease"[Title/Abstract])) OR ("Bechterew's Disease"[Title/Abstract])) OR ("Bechterews Disease"[Title/Abstract])) OR ("Marie-Struempell Disease"[Title/Abstract])) OR ("Marie Struempell Disease"[Title/Abstract])) OR ("Rheumatoid Spondylitis"[Title/Abstract])) OR (spondylitis,[Title/Abstract] AND rheumatoid[Title/Abstract])) OR ("Spondylitis Ankylopoietica"[Title/Abstract])) OR ("Ankylosing Spondyloarthrit*"[Title/Abstract])) OR (spondyloarthrit*,[Title/Abstract] AND ankylosing[Title/Abstract])) OR ("Juvenile Arthrit*"[Title/Abstract])) OR (arthrit*, "Juvenile Chronic"[Title/Abstract])) OR ("Chronic Arthrit*" ,[Title/Abstract] AND juvenile[Title/Abstract])) OR ("Juvenile Rheumatoid Arthrit*"[Title/Abstract])) OR (arthrit*, "Juvenile Idiopathic"[Title/Abstract])) OR ("Juvenile Chronic Arthrit*"[Title/Abstract])) OR (arthrit*, "Juvenile Rheumatoid"[Title/Abstract])) OR ("Rheumatoid Arthrit*" ,[Title/Abstract] AND juvenile[Title/Abstract])) OR ("Juvenile Idiopathic Arthrit*"[Title/Abstract])) OR ("Idiopathic Arthrit*" ,[Title/Abstract] AND juvenile[Title/Abstract])) OR ("Juvenile-Onset Still Disease"[Title/Abstract])) OR ("Juvenile Onset Still Disease"[Title/Abstract])) OR ("Still's Disease" , "Juvenile-Onset"[Title/Abstract])) OR ("Juvenile-Onset Still's Disease"[Title/Abstract])) OR ("Still's Disease" , "Juvenile Onset"[Title/Abstract])) OR ("Still Disease" , "Juvenile-Onset"[Title/Abstract])) OR ("Still Disease" , "Juvenile Onset"[Title/Abstract])) OR ("Systemic Arthrit*" ,[Title/Abstract] AND juvenile[Title/Abstract])) OR (arthrit*, "Juvenile Systemic"[Title/Abstract])) OR ("Juvenile Systemic Arthrit*"[Title/Abstract])) OR ("Juvenile-Onset Stills Disease"[Title/Abstract])) OR ("Juvenile Onset Stills Disease"[Title/Abstract])) OR ("Stills Disease" , "Juvenile-Onset"[Title/Abstract])) OR (polyarthrit*,[Title/Abstract] AND juvenile,

	"Rheumatoid Factor Positive"[Title/Abstract])) OR (polyarthrit*,[Title/Abstract] AND juvenile, "Rheumatoid Factor Negative"[Title/Abstract])) OR (oligoarthrit*,[Title/Abstract] AND juvenile[Title/Abstract])) OR ("Juvenile Oligoarthrit*"[Title/Abstract])) OR ("Enthesitis-Related Arthrit*" ,[Title/Abstract] AND juvenile[Title/Abstract])) OR (arthrit*, "Juvenile Enthesitis-Related"[Title/Abstract])) OR ("Enthesitis Related Arthrit*" ,[Title/Abstract] AND juvenile[Title/Abstract])) OR ("Juvenile Enthesitis-Related Arthrit*"[Title/Abstract])) OR ("Psoriatic Arthrit*" ,[Title/Abstract] AND juvenile[Title/Abstract])) OR (arthrit*, "Juvenile Psoriatic"[Title/Abstract])) OR (arthrit*,[Title/Abstract] AND gouty[Title/Abstract])) OR ("Gouty Arthrit*"[Title/Abstract])) OR (arthrit*,[Title/Abstract] AND reactive[Title/Abstract])) OR ("Reactive Arthrit*"[Title/Abstract])) OR (arthrit*, "Post-Infectious"[Title/Abstract])) OR (arthrit*, "Post Infectious"[Title/Abstract])) OR ("Post-Infectious Arthrit*"[Title/Abstract])) OR ("Post Infectious Arthrit*"[Title/Abstract])) OR ("Postinfectious Arthrit*"[Title/Abstract])) OR (arthrit*,[Title/Abstract] AND postinfectious[Title/Abstract])) OR ("Reiter Syndrome"[Title/Abstract])) OR ("Reiter's Disease"[Title/Abstract])) OR ("Reiters Disease"[Title/Abstract])) OR ("Reiter's Syndrome"[Title/Abstract])) OR ("Reiters Syndrome"[Title/Abstract])) OR ("Reiter Disease"[Title/Abstract])) OR (arthrit*,[Title/Abstract] AND reactive[Title/Abstract])) OR ("Infectious Arthrit*"[Title/Abstract])) OR (arthrit*,[Title/Abstract] AND viral[Title/Abstract])) OR ("Viral Arthrit*"[Title/Abstract])) OR (arthrit*,[Title/Abstract] AND bacterial[Title/Abstract])) OR (arthrit*,[Title/Abstract] AND septic[Title/Abstract])) OR ("Septic Arthrit*"[Title/Abstract])) OR ("Bacterial Arthrit*"[Title/Abstract])) OR (arthrit*,[Title/Abstract] AND suppurative[Title/Abstract])) OR ("Suppurative Arthrit*"[Title/Abstract])) OR (arthrit*,[Title/Abstract] AND infectious[Title/Abstract])) OR ("Enteropathic arthrit*"[Title/Abstract])) OR ("Traumatic arthrit*"[Title/Abstract])) OR (psoriasis,[Title/Abstract] AND arthritic[Title/Abstract])) OR ("Arthritic Psoriasis"[Title/Abstract])) OR ("Psoriatic Arthritis"[Title/Abstract])) OR ("Psoriasis Arthropathica"[Title/Abstract])) OR ("Psoriatic Arthropathy"[Title/Abstract])) OR (arthropath*,[Title/Abstract] AND psoriatic[Title/Abstract])) OR ("Psoriatic Arthropath*"[Title/Abstract])) OR (arthrit*,[Title/Abstract] AND psoriatic[Title/Abstract])) OR (arthrit*[Title/Abstract])) OR (polyarthrit*[Title/Abstract]) AND ((((((((curcumin[MeSH Terms]) OR (curcumin*[Title/Abstract])) OR (curcuma[Title/Abstract])) OR (turmeric*[Title/Abstract])) OR ("Curcuma zedoaria"[Title/Abstract])) OR (zedoaria*,[Title/Abstract] AND curcuma*[Title/Abstract])) OR ("Zedoary zedoaria"[Title/Abstract])) OR (zedoaria*,[Title/Abstract] AND zedoary[Title/Abstract])) OR ("Curcuma longa*"[Title/Abstract])) OR (longa*,[Title/Abstract] AND curcuma[Title/Abstract])
**Scopus**	(TITLE-ABS-KEY (nanoparticle*) OR TITLE-ABS-KEY (nanocapsule*) OR TITLE-ABS-KEY (nanogel*) OR TITLE-ABS-KEY (nanosphere*) OR TITLE-ABS-KEY ("Nanoparticle Drug Delivery System*") OR TITLE-ABS-KEY ("Drug carrier*") OR TITLE-ABS-KEY ("Solid Lipid Nanoparticle*") OR TITLE-ABS-KEY ("Nanostructured lipid carrier*") OR TITLE-ABS-KEY (nanoemulsion*) OR TITLE-ABS-KEY (microemulsion*) OR TITLE-ABS-KEY (nanocarrier*)

OR TITLE-ABS-KEY ("Lipid nanoparticle*") OR TITLE-ABS-KEY ("Nanostructured system*") OR TITLE-ABS-KEY (liposome*) OR TITLE-ABS-KEY (ethosome*) OR TITLE-ABS-KEY (niosome*) OR TITLE-ABS-KEY (cyclodextrin*)) AND (TITLE-ABS-KEY ("Rheumatoid arthrit") OR TITLE-ABS-KEY (arthrit*, AND rheumatoid) OR TITLE-ABS-KEY (osteoarthrit*) OR TITLE-ABS-KEY (arthrit*, AND degenerative) OR TITLE-ABS-KEY ("Degenerative Arthrit*") OR TITLE-ABS-KEY ("Osteoarthros* Deformans") OR TITLE-ABS-KEY ("Spondyloarthrit* Ankylopoietica") OR TITLE-ABS-KEY ("Ankylosing Spondylarthrit*") OR TITLE-ABS-KEY (spondylarthrit*, AND ankylosing) OR TITLE-ABS-KEY ("Ankylosing Spondylitis") OR TITLE-ABS-KEY ("Spondylarthrit* Ankylopoietica") OR TITLE-ABS-KEY ("Bechterew Disease") OR TITLE-ABS-KEY ("Bechterew's Disease") OR TITLE-ABS-KEY ("Bechterews Disease") OR TITLE-ABS-KEY ("Marie-Struempell Disease") OR TITLE-ABS-KEY ("Marie Struempell Disease") OR TITLE-ABS-KEY ("Rheumatoid Spondylitis") OR TITLE-ABS-KEY (spondylitis, AND rheumatoid) OR TITLE-ABS-KEY ("Spondylitis Ankylopoietica") OR TITLE-ABS-KEY ("Ankylosing Spondyloarthrit*") OR TITLE-ABS-KEY (spondyloarthrit*, AND ankylosing) OR TITLE-ABS-KEY ("Juvenile Arthrit*") OR TITLE-ABS-KEY (arthrit*, "Juvenile Chronic") OR TITLE-ABS-KEY ("Chronic Arthrit*" , AND juvenile) OR TITLE-ABS-KEY ("Juvenile Rheumatoid Arthrit*") OR TITLE-ABS-KEY (arthrit*, "Juvenile Idiopathic") OR TITLE-ABS-KEY ("Juvenile Chronic Arthrit*") OR TITLE-ABS-KEY (arthrit*, "Juvenile Rheumatoid") OR TITLE-ABS-KEY ("Rheumatoid Arthrit*" , AND juvenile) OR TITLE-ABS-KEY ("Juvenile Idiopathic Arthrit*") OR TITLE-ABS-KEY ("Idiopathic Arthrit*" , AND juvenile) OR TITLE-ABS-KEY ("Juvenile-Onset Still Disease") OR TITLE-ABS-KEY ("Juvenile Onset Still Disease") OR TITLE-ABS-KEY ("Still's Disease" , "Juvenile-Onset") OR TITLE-ABS-KEY ("Juvenile-Onset Still's Disease") OR TITLE-ABS-KEY ("Still's Disease" , "Juvenile Onset") OR TITLE-ABS-KEY ("Still Disease" , "Juvenile-Onset") OR TITLE-ABS-KEY ("Still Disease" , "Juvenile Onset") OR TITLE-ABS-KEY ("Systemic Arthrit*" , AND juvenile) OR TITLE-ABS-KEY (arthrit*, "Juvenile Systemic") OR TITLE-ABS-KEY ("Juvenile Systemic Arthrit*") OR TITLE-ABS-KEY ("Juvenile-Onset Stills Disease") OR TITLE-ABS-KEY ("Juvenile Onset Stills Disease") OR TITLE-ABS-KEY ("Stills Disease" , "Juvenile-Onset") OR TITLE-ABS-KEY (polyarthrit*, AND juvenile, "Rheumatoid Factor Positive") OR TITLE-ABS-KEY (polyarthrit*, AND juvenile, "Rheumatoid Factor Negative") OR TITLE-ABS-KEY (oligoarthrit*, AND juvenile) OR TITLE-ABS-KEY ("Juvenile Oligoarthrit*") OR TITLE-ABS-KEY ("Enthesitis-Related Arthrit*" , AND juvenile) OR TITLE-ABS-KEY (arthrit*, "Juvenile Enthesitis-Related") OR TITLE-ABS-KEY ("Enthesitis Related Arthrit*" , AND juvenile) OR TITLE-ABS-KEY ("Juvenile Enthesitis-Related Arthrit*") OR TITLE-ABS-KEY ("Psoriatic Arthrit*" , AND juvenile) OR TITLE-ABS-KEY (arthrit*, "Juvenile Psoriatic") OR TITLE-ABS-KEY (arthrit*, AND gouty) OR TITLE-ABS-KEY ("Gouty Arthrit*") OR TITLE-ABS-KEY (arthrit*, AND reactive) OR TITLE-ABS-KEY ("Reactive Arthrit*") OR TITLE-ABS-KEY (arthrit*, "Post-Infectious") OR TITLE-ABS-KEY (arthrit*, "Post Infectious") OR TITLE-ABS-KEY ("Post-Infectious Arthrit*") OR TITLE-ABS-KEY ("Post

	Infectious Arthrit*") OR TITLE-ABS-KEY ("Postinfectious Arthrit*") OR TITLE-ABS-KEY (arthrit*, AND postinfectious) OR TITLE-ABS-KEY ("Reiter Syndrome") OR TITLE-ABS-KEY ("Reiter's Disease") OR TITLE-ABS-KEY ("Reiters Disease") OR TITLE-ABS-KEY ("Reiter's Syndrome") OR TITLE-ABS-KEY ("Reiters Syndrome") OR TITLE-ABS-KEY ("Reiter Disease") OR TITLE-ABS-KEY (arthrit*, AND reactive) OR TITLE-ABS-KEY ("Infectious Arthrit*") OR TITLE-ABS-KEY (arthrit*, AND viral) OR TITLE-ABS-KEY ("Viral Arthrit*") OR TITLE-ABS-KEY (arthrit*, AND bacterial) OR TITLE-ABS-KEY (arthrit*, AND septic) OR TITLE-ABS-KEY ("Septic Arthrit*") OR TITLE-ABS-KEY ("Bacterial Arthrit*") OR TITLE-ABS-KEY (arthrit*, AND suppurative) OR TITLE-ABS-KEY ("Suppurative Arthrit*") OR TITLE-ABS-KEY (arthrit*, AND infectious) OR TITLE-ABS-KEY ("Enteropathic arthrit*") OR TITLE-ABS-KEY ("Traumatic arthrit*") OR TITLE-ABS-KEY (psoriasis, AND arthritic) OR TITLE-ABS-KEY ("Arthritic Psoriasis") OR TITLE-ABS-KEY ("Psoriatic Arthritis") OR TITLE-ABS-KEY ("Psoriasis Arthropathica") OR TITLE-ABS-KEY ("Psoriatic Arthropathy") OR TITLE-ABS-KEY (arthropath*, AND psoriatic) OR TITLE-ABS-KEY ("Psoriatic Arthropath*") OR TITLE-ABS-KEY (arthrit*, AND psoriatic) OR TITLE-ABS-KEY (arthrit*) OR TITLE-ABS-KEY (polyarthrit*)) AND (TITLE-ABS-KEY (curcumin*) OR TITLE-ABS-KEY (curcuma) OR TITLE-ABS-KEY (turmeric*) OR TITLE-ABS-KEY ("Curcuma zedoaria") OR TITLE-ABS-KEY (zedoaria*, AND curcuma*) OR TITLE-ABS-KEY ("Zedoary zedoaria") OR TITLE-ABS-KEY (zedoaria*, AND zedoary) OR TITLE-ABS-KEY ("Curcuma longa*") OR TITLE-ABS-KEY (longa*, AND curcuma))
**Web of Science**	TS=(nanoparticle*) OR TS=(nanocapsule*) OR TS=(nanogel*) OR TS=(nanosphere*) OR TS=("Nanoparticle Drug Delivery System*") OR TS=("Drug carrier*") OR TS=("Solid Lipid Nanoparticle*") OR TS=("Nanostructured lipid carrier*") OR TS=(nanoemulsion*) OR TS=(microemulsion*) OR TS=(nanocarrier*) OR TS=("Lipid nanoparticle*") OR TS=("Nanostructured system*") OR TS=(liposome*) OR TS=(ethosome*) OR TS=(niosome*) OR TS=(cyclodextrin*) AND TS=("Rheumatoid arthrit*") OR TS=(Arthrit*, Rheumatoid) OR TS=(Osteoarthrit*) OR TS=(Arthrit*, Degenerative) OR TS=("Degenerative Arthrit*") OR TS=("Osteoarthros* Deformans") OR TS=("Spondyloarthrit* Ankylopoietica") OR TS=("Ankylosing Spondylarthrit*") OR TS=(Spondylarthrit*, Ankylosing) OR TS=("Ankylosing Spondylitis") OR TS=("Spondylarthrit* Ankylopoietica") OR TS=("Bechterew Disease") OR TS=("Bechterew's Disease") OR TS=("Bechterews Disease") OR TS=("Marie-Struempell Disease") OR TS=("Marie Struempell Disease") OR TS=("Rheumatoid Spondylitis") OR TS=(Spondylitis, Rheumatoid) OR TS=("Spondylitis Ankylopoietica") OR TS=("Ankylosing Spondyloarthrit*") OR TS=(Spondyloarthrit*, Ankylosing) OR TS=("Juvenile Arthrit*") OR TS=(Arthrit*, "Juvenile Chronic") OR TS=("Chronic Arthrit*", Juvenile) OR TS=("Juvenile Rheumatoid Arthrit*") OR TS=(Arthrit*, "Juvenile Idiopathic") OR TS=("Juvenile Chronic Arthrit*") OR TS=(Arthrit*, "Juvenile Rheumatoid") OR TS=("Rheumatoid Arthrit*", Juvenile) OR TS=("Juvenile Idiopathic Arthrit*") OR TS=("Idiopathic

	Arthrit*", Juvenile) OR TS=("Juvenile-Onset Still Disease") OR TS=("Juvenile Onset Still Disease") OR TS=("Still's Disease", "Juvenile-Onset") OR TS=("Juvenile-Onset Still's Disease") OR TS=("Still's Disease", "Juvenile Onset") OR TS=("Still Disease", "Juvenile-Onset") OR TS=("Still Disease", "Juvenile Onset") OR TS=("Systemic Arthrit*", Juvenile) OR TS=(Arthrit*, "Juvenile Systemic") OR TS=("Juvenile Systemic Arthrit*") OR TS=("Juvenile-Onset Stills Disease") OR TS=("Juvenile Onset Stills Disease") OR TS=("Stills Disease", "Juvenile-Onset") OR TS=(Polyarthrit*, Juvenile, "Rheumatoid Factor Positive") OR TS=(Polyarthrit*, Juvenile, "Rheumatoid Factor Negative") OR TS=(Oligoarthrit*, Juvenile) OR TS=("Juvenile Oligoarthrit*") OR TS=("Enthesitis-Related Arthrit*", Juvenile) OR TS=(Arthrit*, "Juvenile Enthesitis-Related") OR TS=("Enthesitis Related Arthrit*", Juvenile) OR TS=("Juvenile Enthesitis-Related Arthrit*") OR TS=("Psoriatic Arthrit*", Juvenile) OR TS=(Arthrit*, "Juvenile Psoriatic") OR TS=(Arthrit*, Gouty) OR TS=("Gouty Arthrit*") OR TS=(Arthrit*, Reactive) OR TS=("Reactive Arthrit*") OR TS=(Arthrit*, "Post-Infectious") OR TS=(Arthrit*, "Post Infectious") OR TS=("Post-Infectious Arthrit*") OR TS=("Post Infectious Arthrit*") OR TS=("Postinfectious Arthrit*") OR TS=(Arthrit*, Postinfectious) OR TS=("Reiter Syndrome") OR TS=("Reiter's Disease") OR TS=("Reiters Disease") OR TS=("Reiter's Syndrome") OR TS=("Reiters Syndrome") OR TS=("Reiter Disease") OR TS=(Arthrit*, Reactive) OR TS=("Infectious Arthrit*") OR TS=(Arthrit*, Viral) OR TS=("Viral Arthrit*") OR TS=(Arthrit*, Bacterial) OR TS=(Arthrit*, Septic) OR TS=("Septic Arthrit*") OR TS=("Bacterial Arthrit*") OR TS=(Arthrit*, Suppurative) OR TS=("Suppurative Arthrit*") OR TS=(Arthrit*, Infectious) OR TS=("Enteropathic arthrit*") OR TS=("Traumatic arthrit*") OR TS=(Psoriasis, Arthritic) OR TS=("Arthritic Psoriasis") OR TS=("Psoriatic Arthritis") OR TS=("Psoriasis Arthropathica") OR TS=("Psoriatic Arthropathy") OR TS=(Arthropath*, Psoriatic) OR TS=("Psoriatic Arthropath*") OR TS=(Arthrit*, Psoriatic) OR TS=(Arthrit*) OR TS=(Polyarthrit*) AND TS=(Curcumin*) OR TS=(Curcuma) OR TS=(Turmeric*) OR TS=("Curcuma zedoaria") OR TS=(“zedoaria, Curcuma”) OR TS=("Zedoary zedoaria") OR TS=(“zedoaria, Zedoary”) OR TS=("Curcuma longa") OR TS=(“longa, Curcuma”)

Table S2. Initial data organization for creating the Sankey plots. [Excel file]

Table S3. Excluded articles and reasons.

Authors	Article's title	Year of publication	Reason for exclusion
Abbas et al.	PLA-coated Imwitor® 900 K-based herbal colloidal carriers as novel candidates for the intra-articular treatment of arthritis	2021	Co-loading of CUR and another molecule in the same formulation.

Allijn et al.	Comparison of pharmaceutical nanoformulations for curcumin: Enhancement of aqueous solubility and carrier retention	2016	No in-vivo model was applied to evaluate formulation efficacy and safety.
Asif et al.	Synthesis, characterization and evaluation of anti-arthritic and anti-inflammatory potential of curcumin loaded chitosan nanoparticles	2023	No in-vivo model was applied to evaluate formulation efficacy and safety
Baharizade et al.	Revolutionizing Knee Osteoarthritis Treatment: Innovative Self-Nano-Emulsifying Polyethylene Glycol Organogel of Curcumin for Effective Topical Delivery	2023	Crosslink probably changed the original structure of curcumin.
Campos et al.	Synthesis and characterization of gold nanoparticles combined with curcumin and its effect on experimental osteoarthritis in mice	2015	Abstracts from the 2015 World Congress.
Chen et al.	Erythrocyte membrane-camouflaged and double-factor sequential delivery nanocarriers postpone the progression of osteoarthritis	2023	CUR was chemically modified to prepare the nano-based carrier system.
Chen et al.	Carrier-free nanodrug targeting glucose metabolism for enhanced rheumatoid arthritis treatment	2024	The research does not explore nano-based systems for CUR incorporation.
Li et al.	Curcumin-containing PLGA nanoparticle as a carrier for the treatment of osteoarthritis in rabbits OA model	2016	Wrong study type.
Mande et al.	Solid Dispersion of Curcumin as Polymeric Films for Bioenhancement and Improved Therapy of Rheumatoid Arthritis	2016	Not nanoparticle.
Nivetha et al.	Efficacy of Nanocurcumin with application of Iontophoresis on Inflammatory arthritis patients.	2022	Association of CUR-loaded nanocarrier and other interventions.

Saleem et al.	Curcuminoids-enriched extract and its cyclodextrin inclusion complexes ameliorates arthritis in complete Freund's adjuvant-induced arthritic mice via modulation of inflammatory biomarkers and suppression of oxidative stress markers	2023	The research does not explore nano-based systems for CUR incorporation.
Tang et al.	Self-Report Amphiphilic Polymer-Based Drug Delivery System with ROS-Triggered Drug Release for Osteoarthritis Therapy	2023	Co-loading of CUR and another molecule in the same formulation.
Wang et al.	Hyaluronic Acid Modified Curcumin-Loaded Chitosan Nanoparticles Inhibit Chondrocyte Apoptosis to Attenuate Osteoarthritis via Upregulation of Activator Protein 1 and RUNX Family Transcription Factor 2	2022	No retrieved study.
Yang et al.	A Nanomedicine-Enabled Ion-Exchange Strategy for Enhancing Curcumin-Based Rheumatoid Arthritis Therapy	2023	CUR was chemically modified to prepare the nano-based carrier system.
Zhang et al.	A cyclic brush zwitterionic polymer-based pH-responsive nanocarrier-mediated dual drug delivery system with lubrication maintenance for osteoarthritis treatment	2023	Co-loading of CUR and another molecule in the same formulation.
Zhou et al.	Natural product curcumin-based coordination nanoparticles for treating osteoarthritis via targeting Nrf2 and blocking NLRP3 inflammasome	2021	CUR was chemically modified to prepare the nano-based carrier system.

Table S4. SYRCLE's risk of bias evaluation by domains.

/	Sequence generation (Selection bias)	Baseline characteristics (Selection bias)	Allocation concealment (Selection bias)	Random housing (Performance bias)	Blinding (Performance bias)	Random outcome assessment (Detection bias)	Blinding (Detection bias)	Incomplete outcome data (Attrition bias)	Selective outcome reporting (Reporting bias)	Other sources of bias (Other)	Overall
Kumar and Rai, 2012	Unclear	Unclear	Unclear	Unclear	Unclear	Unclear	Unclear	Unclear	Low	Low	Unclear
Arora et al., 2014	Unclear	Unclear	Unclear	Unclear	Unclear	Unclear	Low	Unclear	Low	Low	Unclear
Naz and Ahmad, 2015	Unclear	Unclear	Unclear	Unclear	Unclear	Unclear	Unclear	Unclear	Low	Low	Unclear
Zheng et al., 2015	Unclear	Unclear	Unclear	Unclear	Unclear	Unclear	Unclear	Unclear	Low	Low	Unclear
Jeengar et al., 2016	Unclear	Unclear	Unclear	Unclear	Low	Unclear	Unclear	Unclear	Low	Low	Unclear
Zhang et al., 2016	Unclear	Unclear	Unclear	Unclear	Unclear	Unclear	Low	Unclear	Low	Low	Unclear
Dewangan et al., 2017	Unclear	Unclear	Unclear	Unclear	Unclear	Unclear	Low	Unclear	Low	Low	Unclear
Campos et al., 2017	Unclear	Unclear	Unclear	Unclear	Unclear	Unclear	Low	Unclear	Low	Low	Unclear
Niazvand et al., 2017	Unclear	Unclear	Unclear	Unclear	Unclear	Unclear	Low	Unclear	Low	Low	Unclear
Sun et al., 2018	Unclear	Unclear	Unclear	Unclear	Unclear	Unclear	Low	Unclear	Low	Low	Unclear
Kiyani et al., 2019	Unclear	Unclear	Unclear	Unclear	Unclear	Unclear	Unclear	Unclear	Low	Low	Unclear
Shinde et al., 2020	Unclear	Unclear	Unclear	Unclear	Unclear	Unclear	Unclear	Unclear	Low	Low	Unclear
Kang et al., 2020	Unclear	Unclear	Unclear	Unclear	Unclear	Unclear	Unclear	Unclear	Low	Low	Unclear
Sana et al., 2021	Unclear	Unclear	Low	Unclear	Unclear	Unclear	Low	Unclear	Low	Low	Unclear
Wang et al., 2021	Unclear	Unclear	Unclear	Unclear	Unclear	Unclear	Unclear	Unclear	Low	Low	Unclear
Song et al., 2022	Unclear	Unclear	Unclear	Unclear	Unclear	Unclear	Unclear	Unclear	Low	Low	Unclear
Zhang et al., 2022	Unclear	Unclear	Unclear	Unclear	Unclear	Unclear	Unclear	Unclear	Low	Low	Unclear
Hamdalla et al., 2022	Unclear	Unclear	Unclear	Unclear	Unclear	Unclear	Unclear	Unclear	Low	Low	Unclear
Lin et al., 2023	Unclear	Unclear	Unclear	Unclear	Unclear	Unclear	Unclear	Unclear	Low	Low	Unclear
Wang et al., 2023	Unclear	Unclear	Unclear	Unclear	Unclear	Unclear	Unclear	Unclear	Low	Low	Unclear
Khashan et al., 2023	Unclear	Unclear	Unclear	Unclear	Unclear	Unclear	Unclear	Unclear	Low	Low	Unclear
Hu et al., 2023	Unclear	Unclear	Unclear	Unclear	Unclear	Unclear	Unclear	Unclear	Low	Low	Unclear
Okpalaku et al., 2023	Unclear	Unclear	Unclear	Unclear	Unclear	Unclear	Unclear	Unclear	Low	Low	Unclear
Wang et al. 2024	Unclear	Unclear	Unclear	Unclear	Unclear	Unclear	Unclear	Unclear	Low	Low	Unclear
Javed et al., 2024	Unclear	Unclear	Unclear	Unclear	Unclear	Unclear	Unclear	Unclear	Low	Low	Unclear
Sun et al., 2024	Unclear	Unclear	Unclear	Unclear	Unclear	Unclear	Unclear	Unclear	Low	Low	Unclear
Xie et al., 2024	Unclear	Unclear	Unclear	Unclear	Unclear	Unclear	Unclear	Unclear	Low	Low	Unclear
Xu et al., 2024	Unclear	Unclear	Unclear	Unclear	Unclear	Unclear	Unclear	Unclear	Low	Low	Unclear
Zhang et al., 2024	Unclear	Unclear	Unclear	Unclear	Unclear	Unclear	Low	Unclear	Low	Low	Unclear
Pérez-Expósito et al., 2024	Unclear	Unclear	Unclear	Unclear	Unclear	Unclear	Unclear	Unclear	Low	Low	Unclear
Azeez et al., 2024	Unclear	Unclear	Unclear	Unclear	Unclear	Unclear	Unclear	Unclear	Low	Low	Unclear

Scores of each paper per domain (D): 1 - Sequence generation; 2 - Baseline characteristics; 3 - Allocation concealment; 4 - Random housing; 5 – Blinding; 6 - Random outcome assessment; 7 – Blinding; 8 - Incomplete outcome data; 9 - Selective outcome reporting; 10 - Other sources of bias.
